# An unexpected transition to virtual care: family medicine residents’ experience during the COVID-19 pandemic

**DOI:** 10.1186/s12875-022-01728-5

**Published:** 2022-05-25

**Authors:** Neale Smith, Christie Newton, Demetra Barbacuta, Olivia Ling-I Tseng

**Affiliations:** 1 Centre for Clinical Epidemiology & Evaluation (C2E2), Vancouver Coastal Health Research Institute (VCHRI), 7th floor Research Pavilion, 828 West 10th Avenue, Vancouver, British Columbia V5Z 1M9 Canada; 2 Department of Family Practice, Faculty of Medicine, University of British Columbia (UBC), 3rd floor David Strangway Building, 5950 University Boulevard Building, Vancouver, British Columbia V6T 1Z3 Canada

**Keywords:** Primary care, Residency, Virtual care, Virtual learning, Family medicine, Pandemic, British Columbia, Canada

## Abstract

**Background:**

The global COVID-19 pandemic led to rapid changes in both medical care and medical education, particularly involving the rapid substitution of virtual solutions for traditional face-to-face appointments. There is a need for research into the effects and impacts of such changes. The objective of this article investigates the perspectives of Family Medicine Residents in one university program in order to understand the impact of this transition to virtual care and learning.

**Methods:**

This is a qualitative focus group study. Four focus groups, stratified by site type (Rural = 1; Semi-Urban = 1; Urban = 2) were conducted, with a total of 25 participants. Participants were either first or second-year Residents in Family Medicine. Focus group recordings were analyzed thematically, based upon a five-level socio-ecological model (individual, family, organization, community, environment and policy context).

**Results:**

Two main themes were identified: (1) Residents’ experiences of Virtual Learning and Virtual Care, and (2) Living and Learning in Pandemic Times. In the first theme, Residents reported challenges both individually, in their family context, and in their training organizations. Of particular concern was the loss of hands-on experience with clinical skills such as conducting physical examinations. In the second theme, Residents reported disruption of self-care routines and family life. These Residents were unable to engage in the relationships outside of the workplace with their preceptors and peers which they had expected, and which play key roles in social support as well as in future decisions about practice location.

**Conclusions:**

While many patients appreciated virtual care, in the eyes of these Residents it is not the ideal modality for learning the practice of Family Medicine, and they awaited a return to normal times. Despite this, the pandemic has pointed out important ways in which residency training needs to adapt to an evolving world.

**Supplementary Information:**

The online version contains supplementary material available at 10.1186/s12875-022-01728-5.

## Background

The global COVID-19 pandemic emerged rapidly in early 2020 [1]. In British Columbia (BC), Canada, the first case was detected on January 26th, 2020. Substantial numbers of additional cases followed, leading to the declaration of a public health emergency by the BC provincial government on March 17th, 2020 [2]. Physical distancing, or the maintenance of a safe distance between individuals, was a key public health measure intended to reduce virus transmission [3]. To implement this measure, non-essential services including schools and other public facilities were closed; essential services were transitioned to virtual settings using such alternatives as video conferences, telephone consultations, email, and text messaging.

In this article, we consider the impact of this upon UBC Family Medicine (FM) Residents. The UBC FM Residency Program has approximately 370 Residents (female to male ratio 59:41) and 3500 preceptors -- practicing family physicians who teach and supervise trainees -- distributed across 20 Training Sites in urban, semi-urban and rural regions. Each Training Site accepts four to 24 Residents annually, who have completed their medical degree at a school in Canada or abroad. The residency is two-years in length, combining clinical training with scholarly activities. Primary care is the main clinical component, mixed with multiple blocks of training across other contexts such as maternity care and hospice/palliative care. Each Resident is assigned to a preceptor, or team of preceptors, and delivers longitudinal primary care under supervision at their preceptors’ practices. Training Sites are responsible for block scheduling, monitoring their Residents’ progress and addressing their learning needs. During the pandemic, the Program and Training Sites modified their curriculum with the goal of striking a balance between learning needs, safety and Residents’ well-being.

At the start of the pandemic, the Program transitioned all educational activities to a virtual learning format using video-conferencing. Preceptors, similar to other practicing family physicians who did not teach, adapted to deliver largely virtual care, mixed with limited in-person care. When Residents were scheduled to work at their preceptors’ practices, Residents either provided hybrid virtual and in-person care or they provided solely virtual care usually from their own residences (“working from home”).

How did these changes in training, which were made rapidly and without existing evidence, affect Residents’ learning? To guide future residency training, this study aims to explore FM Residents’ experience with delivering virtual care and participating in virtual learning activities during the COVID-19 pandemic in BC.

## Methods

### Recruitment

As this project was considered to be quality improvement conducted within the UBC Faculty of Medicine (FOM), Department of Family Practice (DFP), formal ethics approval was not sought. Participants were recruited through a three-step process of (i) initial invitation, (ii) scheduling to a focus group, and (iii) confirmation of participation, including distribution of consent forms along with a copy of the focus group guide. An initial invitation with a survey link to participate was distributed to both first- and second-year FM Residents in BC through member-level email lists of the UBC DFP, Divisions of Family Practice (regional professional primary care organizations), and BC Family Doctors (provincial professional association) between March 31st and April 6th, 2021. Resident status was confirmed by cross-referencing their educational licenses published on the registrant directory of the College of Physicians and Surgeons of British Columbia (https://www.cpsbc.ca/public/registrant-directory). The survey collected each Resident’s demographics (as listed in Table 1). A total of 37 expressions of interest were received and stratified by training site setting, either urban, semi-urban or rural; these categories were based on definitions from the UBC DFP (https://carms.familymed.ubc.ca/training-sites/site-comparison/), confirmed with review of population sizes using Statistics Canada data. Four focus groups, corresponding to the training settings, were scheduled on the evenings of April 13th (Semi-Urban), April 14th (Urban 1), April 15th (Rural) and April 20th (Urban 2). Each group was limited to up to eight Residents to ensure all participants’ voices were heard. Urban Residents were invited to participate in either the first or second focus groups based on a first-come, first-served basis. A second email with date and time, based on their Training Site setting and time order of response (for Urban Residents only), was sent to all 37 Residents five to seven days prior to the scheduled focus groups. A total of 26 Residents confirmed their participation, at this time. Six Residents declined because of their schedule conflicts with their on-call days or exams, and five did not respond. A third email with a focus group guide and consent forms (see Additional file 1) were circulated at least one day prior to the meeting. Twenty-five of the 26 confirmed participants joined the scheduled online sessions at the given time.

**Table 1:** Participant demographics

# of participants	Total	Total %
	25	
Gender Identity		
Male	11	44%
Female	14	56%
Equity Group		
Yes	10	40%
No	14	56%
No Response	1	4%
Age Group		
25–29	10	40%
30–34	11	44%
35–39	2	8%
40–44	1	4%
45–49	1	4%
50 or Above	0	
Training Site		
Urban^a^	13	52%
Semi-Urban	7	28%
Rural	5	20%
International Medical Graduate (IMG)		
Yes	10	40%
No	14	56%
No Response	1	4%
Residency Year		
R1	16	64%
R2	9	36%

^a^13 Residents were split among two focus groups (8 and 5 respectively)

### Conduct of the focus groups

Four focus groups with 25 Residents were conducted, using the Zoom platform sponsored by the UBC FOM, between April 13th and 20th, 2021. Participants received an honorarium, in the form of a $50 e-gift card, for attending. The groups were conducted by an experienced qualitative researcher, external to the UBC Residency Program; the groups were recorded with participant consent, and detailed notes were also taken by a research assistant.

### Data analysis

The recordings were reviewed by the focus group moderator. Key ideas were highlighted and categorized into either Themes One or Two, below, corresponding to topics one (virtual learning and virtual care) and two (living and learning in pandemic times). Under each theme, key ideas were further grouped into levels of a socio-ecological system [4] through cycles of iterative discussions among team members with research, clinical practice and teaching expertise. The socio-ecological system included five levels [5, 6]: individual; family/friends (micro); organization, representing preceptors and programs (meso); community, representing patients and population and environmental/system (macro). Specific and relevant quotes made by Residents were then extracted.

## Results

Of the 25 participants, 56% identified as female and 44% as male. 40% of participants self-identified as members of equity groups, and 40% were international medical graduates (IMGs). 64% of participants were in Year I of their residency, and 36% in Year II (See Table 1). Participants came from 12 of the 20 Training Sites across BC.

**Fig. 1:**
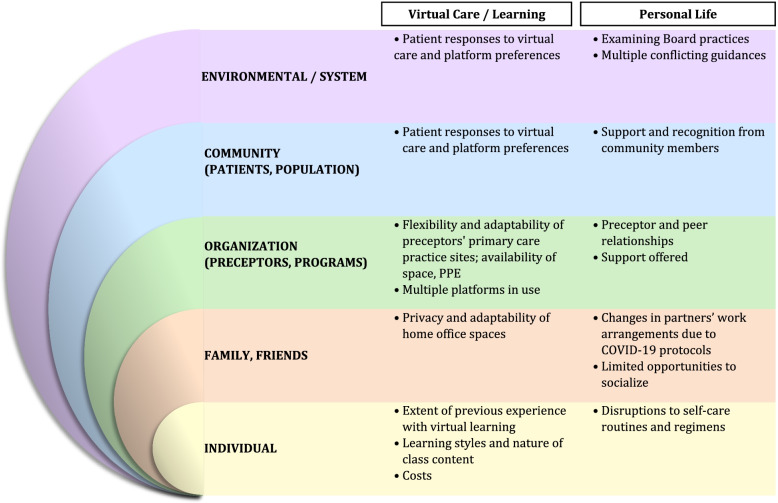
Study findings mapped onto the socio-ecological model. Family medicine Residents’ experiences of virtual learning and virtual care and personal life in pandemic times are summarized

Findings of the qualitative analysis are divided into two main themes: (1) Residents’ Experiences of Virtual Learning and Virtual Care, and (2) Living and Learning in Pandemic Times. Results were considered throughout using a socio-ecological lens. This began with considering organizational factors which influenced residency training. The analysis was broadened to consider relevant influences at the individual and family/interpersonal levels, and then looking outwards to encompass community-level influences and the role of the larger socio-cultural environment. The major points from both Themes are summarized in Fig. 1.

### Theme one -- Residents’ experiences of virtual learning and virtual care

The first theme focuses on the Residents’ scholarly and clinical learning experience during the pandemic. The learning experience was, either positively or negatively, shaped by multi-level factors ranging from individual to community.

#### Individual

At the individual level, participants had extremely limited prior virtual care experience. Their greatest exposure to virtual learning was in viewing pre-recorded lectures offered during medical school. Residents found it difficult to maintain their concentration during virtual lectures: “You get distracted, can’t pay as much attention” (Urban 2). “It’s harder to feel in that work setting, be productive from home – I struggled with paying attention” (Semi-Urban). These comments suggest that interactive virtual learning is preferred over simple virtual lectures, though allowances can be made: “if the people providing the teaching are a little more tech savvy, things can be more engaging.” Using Zoom break out rooms, or alternative interactive sites such as Kahoot, were suggested (Semi-Urban). And maybe some topics simply do not work well in virtual format: “Stop trying to teach people suturing over Zoom” (Urban 2).

Adopting technology is not easy for Residents who are not tech-savvy. Early on, participants noted technical glitches that negatively impacted the learning experience -- microphones not working, difficulty in screen sharing, or participants entering sessions unmuted with background noise. Some also experienced direct financial costs, for having to upgrade or acquire new devices (Urban 2); one suggested that it is hard to both watch a Zoom presentation and take notes on the same device, so multiple ones were needed (Semi-Urban).

#### Family

Virtual learning from home was impacted by Residents’ family situations. As one participant described, during their orientation to residency, “I was sharing space with family members, including small children [so I was keeping my video off] … I got a very stern email saying [this] was very inappropriate” (Urban 2); in other words, this situational obstacle was made into a professionalism issue.

#### Organization

Unsurprisingly Residents’ learning experiences were shaped by organizational-level factors, at the university (UBC FM Residency Program), regional (Training Site) or preceptors’ practice levels.

At the Program level, it was suggested the Program could have provided guidelines to preceptors on how to adapt to virtual learning, noting that they often have variable training as educators (Urban 1). While some preceptors responded quickly to the changing situation, others floundered: “When it first transitioned to [virtual care], a lot of our preceptors didn’t know what to do with us at that point” (Rural). “My preceptor wasn’t sure if it could continue at first, but we were able to navigate around it to get tech support that was needed” (Urban 2).

Respondents generally found the Training Sites “flexible and accommodating” (Urban 1; Semi-Urban). For instance, Residents were allowed in some cases to change scheduled rotations (Rural). The Sites also worked to find or offer additional opportunities to practice and hone skills. One Resident noted that, “I was only able to do four or five shifts on Peds” but later the Site Director reached out to say that more sessions would be made available -- “they are trying their best to get us more experience” (Rural). “[Training Site] has made some pretty exceptional effort to fill the holes when they do come up” (Semi-Urban). Moving between clinics and block rotations also posed the frequent challenges of having to learn multiple different technologies and platforms (Rural). “If you move to a different clinic, you have to learn a whole new set up” (Urban 2).

At the Preceptors’ Practice level, Residents also encountered factors which impacted their clinical learning experience. Some respondents noted that, “preceptors tried their best to schedule their interesting patients for those days” when Residents were on site. However, a shortage of personal protective equipment (PPE) meant that Residents were asked repeatedly, “would you mind just sitting this one out?” (Semi-Urban). Some communication apps required clinics to purchase individual user licenses, and Residents were not always provided these (Semi-Urban). Without such user privileges, some Residents were left “sitting in the back of the room listening to them on the phone, which is useless” (Rural). With the above limitations, Residents might be assigned with activities such as prescription renewals which some did not find educationally useful: “Sometimes I would spend all day doing add-ons, 15-minute phone calls one after another, refilling prescriptions —at end of day, I felt like I “filled a need” but learned nothing” (Rural).

These organizational factors, by consensus across all four groups, lead to certain learning gaps. Residents’ clinical decision-making suffered when non-verbal cues were either missing or limited during virtual appointments. “In person, you can look at behaviors, how someone walks into a room, if they have a resting tremor …subtle signs which help you craft a diagnosis and plan” (Urban 1). Without physically seeing patients, the Resident cannot get the “subjective gestalt” (Semi-Urban). Residents reported mixed experience when triaging patients with newly onset symptoms and when discerning which patients needed to be seen in-person immediately, and which could be managed virtually as outpatients (Rural; Urban 1; Urban 2). Some reported improved triaging skills with high-volume virtual appointments while others lowered their thresholds for in-person examinations: “If it’s on the phone, I lean towards sending people to the emerg more often” (Rural); “you end up saying, come in and see me any way” (Urban 2).

Certain clinical procedural and examination skills that could not be done virtually were also highlighted. “I think I did 2 Pap smears this entire year of residency” (Urban 1). “I could count on one hand the number of Pap smears I’ve done in four blocks of Family Medicine” (Urban 2). Skin conditions too, were challenging to diagnose when (often low quality) photos or images proved to be inadequate substitutes for in-person examination (Semi-Urban, Urban 1, Urban 2). In such scenarios, Residents were conflicted: “I have an urge to examine, [yet] feel guilty constantly in asking my preceptor if we can squeeze this patient in, [I] feel guilty about wanting to examine patients in person” (Semi-Urban). Obtaining clinical skills was not a concern for Residents whose preceptors prioritized in-person appointments over virtual appointments for them (Urban 1). Together with their first-year training prior to the pandemic, some second-year Residents did feel confident in their clinical skills (Urban 1).

Another competency noted by participants was time management. Residents seemed to find virtual appointments relatively easy to complete on time, but found that for in-person appointments, it took longer to deal with similar presentations (Urban 1). Multi-tasking --here, the ability to use additional online resources during a virtual appointment in order to maximize outputs -- was a skill which virtual care seemed to have improved for many (Rural; Semi-Urban; Urban 2).

Finally, clinical independence to make decisions alone is another theme which appears across focus groups in this study. For some Residents, virtual care provided them with earlier opportunities to exercise their own judgment; Residents got to “do a few visits by phone at a time without breaks, then review it all with the physician” (Rural). Residents in some sites could also exercise discretion about which patients they needed to bring in for in-person appointments (Urban 1). However, one participant expressed their experience this way: “[It was] hard to take ownership of my patients … [I wasn’t able to call them in as easily] so I have to turn to my preceptor to reassure myself” (Semi-Urban).

#### Community

Community influences were felt through the interactions which Residents had with their patients. Participants noted, across focus groups, their perception that patients felt certain advantages of virtual care. Care became more accessible, for instance to those for whom even prepping to leave the house is a big task (Rural), or for some pediatric patients: “Some kids are much more comfortable at home on the sofa with their blankie” than in the physician’s office (Rural). Patients were also happier not having to spend time traveling to appointments (Rural). Some participants also believed that virtual care was good for continuity of care: “some patients have better attendance for follow-up” (Urban 2).

A common experience was that many patients also preferred the telephone over video platforms for their virtual appointments (Rural). It was described how, in the beginning, clinics and patients would often fumble with video technology before resorting to the telephone. “My practice has given up on video calls because it causes so much problems, only phone calls” (Urban 1). “Started with video in our clinic but it went by the wayside” (Urban 1). Sometimes, the clinics themselves simply had the phone and no video platform at all (Semi-Urban, Urban 2).

### Theme two -- living and learning in pandemic times

The second theme focuses on the Residents’ daily life experience during the pandemic and how those experiences impacted their ability to learn. Participants mostly shared life experiences that had negative impacts on learning; however, some also described valued supports.

#### Individual

At the individual or personal level, participants reported disruption to their self-care routines due to physical distancing requirements and other pandemic-related restrictions, as well as the establishment of new means to maintain work-life balance. For instance, gyms were closed to for periods of time, and respondents felt that absence: “Home workouts I don’t enjoy as much” (Urban 1). “A million home workouts are just not the same” (Rural). On the other hand, outdoor activities remained available. “We felt justified in buying a season’s pass to the ski hill, it’s the only thing we can do” (Semi-Urban).

#### Family

Partners and immediate family were important resources for many: “The biggest thing was my family” (Urban 1). For some, working from home arrangements made their partners more available than had been anticipated. Others reported that their children’s school and out-of-school arrangements were thrown into flux; one participant noted, for instance, “I was unable to get help from family with babysitting” (Urban 1).

Being separated from extended family was also a challenge. One participant noted that they had chosen their residency site specifically because they had family in that community, but under pandemic restrictions, “we weren’t able to be as much part of each other’s lives as planned” (Rural). Also, many Residents were newcomers to BC or to their Training Sites, so in addition to lacking family connections, they found it challenging to meet new people and make friends (Semi-Urban). Certain fears, justified or otherwise, made this difficult: “People you know who aren’t in medicine are scared to meet with you, they don’t want you to come around with these potential viruses” (Urban 1).

#### Organization

Participants were aware of a range of personal supports provided by the University, such as individual or couples counselling, and felt these were well-communicated, though Residents in the focus groups had not had occasion to use these themselves (Urban 2).

A particularly important loss was the opportunity to develop deeper relationships. This begins with preceptors. “My preceptor said, normally I’d have you over for dinner when you first got here, but with COVID that’s not going to happen” (Semi-Urban). “We missed the usual Christmas parties with preceptors and families, getting to know them outside clinic” (Rural).

It includes peers as well. “Peer-to-peer connections among Residents” is one of the ways you get through residency” (Urban 2). “In order to build a good Resident group, you need to know people, connect with people” (Urban 1). Pandemic restrictions punched a hole in one of the safety nets that Residents expected to be available to them: “We don’t really know each other as well on the personal level, so it’s a little harder to reach out if you’re struggling, if you’ve had a bad day and want to talk to someone” (Urban 1). In other words, the Resident cohort never became a “functioning social group” (Rural) in the way many had expected.

#### Community

Community support for the Residents, as health care providers, was observed and welcomed. “The community has made it very clear that they appreciate us” (Rural), evidenced for instance through discounts offered by local restaurants and stores. Nonetheless, some found it isolating -- “I was out there on my own in a small town” (Rural).

#### Environment

Finally, some Residents experienced uncertainty, challenges and frustrations related to the larger policy context and external factors beyond the UBC FM Program. Our focus groups were conducted right before the Medical Council of Canada Part II exam week. One rural Resident perceived this process as “really mis-managed (Rural), for instance in the last-minute cancellation of scheduled exams, often after Residents had arranged time off and booked flights and accommodations (Urban 2). Residents received multiple guidances and direction sources such as the College of Family Physicians of Canada, UBC as an institution, health authorities, and various levels of government (Semi-Urban). Such guidances were not always consistent or easy to follow, and frequent changes bred confusion.

## Discussion

This study documented Residents’ one-year learning journey in BC during the early pandemic era using a focus group approach; this qualitative approach contrasts with cross-sectional surveys elsewhere -- the US and Turkey – yet provides consistent findings [7–9]. The results described how Residents’ learning was affected by inter-linked factors arising in the micro-, meso- and macro-levels. The report shared common interests with other studies on Residents’ clinical activities [7, 9], educational training [7] and personal well-being [7, 8]. From the setting perspective, this study focused on Residents’ learning experience in primary care settings, specifically in their preceptors’ practices, in contrast to overall learning experiences across various settings in other studies [7–9].

The exigencies of the pandemic required that residency training be transformed, practically overnight, from in-person to virtual. FM Residents had no option in this regard. Their learning and care delivery are intimately connected, which are influenced by interrelated factors captured in this study. At the individual level, the Residents had to learn, work and live differently, but this was within a context shaped by community, organizational, environmental and policy factors. The Residents, similar to other healthcare providers, are at a higher risk of psychological stress. Learning to become a physician during the pandemic meant putting their personal health at stake when caring for patients infected with a highly contagious virus [10]. Consistent with experience elsewhere, Residents’ social support further decreased when avoiding social events with friends and family members to reduce the likelihood of transmitting a virus potentially caught from their workplaces [11].

At the organization level, focus group participants felt that the UBC FM Residency Program largely well delivered on the changes, though they provided several suggestions and reflections for improvement. Clinical skills, for the most part, continued to be built. Hands-on direct patient care, a key part of residency training, is missing in the virtual care setting. In the eyes of these Residents, virtual care is not an ideal learning modality when it is challenging to make clinical decisions without input from in-person physical examinations including non-verbal cues [12].

Picking up non-verbal cues is at least equally important to verbal cues, enabling physicians to exercise clinical judgment including intuition and gut feeling to diagnose the ‘gestalt’ of the patient [13]. It remains a question how virtual care and learning will impact this aspect of the COVID Cohort’s clinical decision-making skills. Will these physicians practice differently? —for instance, will they be more or less likely to triage their patients to emergency department care?

Communities were supportive, but it was harder to meet neighbors socially and to build relationships. It remains a question as to how this may impact recruitment of new physicians into smaller, more remote communities. We know from the literature that educational experience in rural locations leads new graduates to be more likely to practice in these communities [14]—will the truncated social experiences from the pandemic year weaken this pull? In short, where will these graduates choose to practice, and how has COVID-19 shaped those choices?

At the environment and policy level, many relevant issues were voiced. While patients themselves in many ways appreciated this new way of business, the province struggled to build the infrastructure to support providers [15], as did the licensing bodies whose exams the Residents must pass in order to enter practice independently [16]. We can envision additional relevant issues that were not identified by our participants, such as regulatory issues which might occur when providing virtual care under provincial medical licenses to patients who are not physically resident in BC.

Based on these findings**,** recognizing the likelihood that virtual learning and virtual care have become established practices which will continue in some form after the pandemic, we would put forward the following recommendations:

1. Canadian residency programs should collaborate with professional bodies to establish educational standards for virtual learning and develop and implement evidence-based virtual care training curricula for both Family Medicine Residents and preceptors.
2. Notwithstanding the above, residency programs must ensure that Residents have adequate exposure to the in-person, hands-on training required to master key Family Medicine competencies. To do so, it may be needful to explore the possibility of extending the FM residency beyond the current two-year length in Canada. This is not striking into entirely uncharted terrain as many jurisdictions have already lengthier programs than Canada [17]. In addition to a longer residency, another option is to add more enhanced skills programs (perhaps 3–13 months in length), which could be offered as an extension to training or as re-entry training for practicing family physicians. In any case, proper resource support will be required.
3. Governments and educational institutions must invest in the supports required to build out and sustain virtual care infrastructure, including supports for primary care practices such as new billing codes for virtual care, and capital costs related to adopting new technologies and where needed, adapting physical spaces to allow for privacy and physical distancing.

The ‘Covid Cohort’ of FM Residents seems likely to become one of the most researched groups of health professionals in recent history. Their training experience has been like no other. There can be much learned by following their entry into practice to see if their experiences are systematically different from those who preceded and those who will follow them. Family physicians in Canada are committed to lifelong learning to maintain their competency after completing their residency training [18]. In the near term, it would be interesting to see if this group disproportionally seeks out additional training through learning events, such as Continuing Professional Development (CPD) activities.

### Strengths and limitations

Our narrower focus allowed us to delve deeper to learn the impact of the COVID pandemic on teaching and learning of essential clinical skills, such as clinical independence and decision making. The advantages this study adopting a focus-group approach are collecting time-sensitive information quickly at a relatively low cost while broadly exploring factors related to a new topic. However, a focus group approach collects information qualitatively, which prevents the team quantifying the impacts of each identified factor. While there was good diversity among participants in terms of variables such as gender, year of residency, and site, there may be other unidentified factors on which participants were not representative of their cohorts; for instance, focus groups were conducted via Zoom and those who had negative experiences or were exhausted with virtual activities may have been less likely to consider participating. Afterhours timing of focus groups may discourage Residents who were on-call or needed to participate in family activities. As in all qualitative research, the results should be interpreted in context, and the BC experience may not reflect that of medical Residents in other schools and provinces in Canada or elsewhere.

## Conclusions

While many patients appreciated virtual care, in the eyes of these Residents it is not the ideal modality for learning the practice of Family Medicine. Hands-on direct patient care, a key part of residency training, is missing in the virtual care settings. Despite Residents’ anticipating a return to normal times, the pandemic has pointed out important ways in which Residency training needs to adapt to an evolving world.

## Supplementary Information

**Additional file 1.** Consent form for study participants.

## Data Availability

To ensure the anonymity of participants, the qualitative data collected in this study cannot be made publically available. Data may be made available from the corresponding author upon request.

## Abbreviations

C2E2: Centre for Clinical Epidemiology & Evaluation
CPD: Continuing professional development
DFP: Department of Family Practice
FM: Family Medicine
FOM: Faculty of Medicine
IMG: International medical graduate
UBC: University of British Columbia
VCHRI: Vancouver Coastal Health Research Institute
