# Supplementary material for: An unexpected transition to virtual care: family medicine residents’ experience during the COVID-19 pandemic
Source: BMC Prim Care. 2022 May 25;23:127. doi: 10.1186/s12875-022-01728-5 (PMC9130052; doi:10.1186/s12875-022-01728-5)
Supplement: Supplementary file 1 — Additional file 1. Consent form for study participants. [file 12875_2022_1728_MOESM1_ESM.pdf]

THE UNIVERSITY OF BRITISH COLUMBIA

Informed Consent

FOCUS GROUP CONSENT FORM

Residents' learning experience during the pandemic

Principal Investigator: Dr. L. Olivia Tseng, UBC (Email: olivia.tseng@ubc.ca)

Co-Principal Investigator: Dr. Christie Newton, UBC (Email: christie.newton@familymed.ubc.ca)

Funder: The College of Family Physicians of Canada

This consent form, a copy of which has been given to you, is only part of the process of informed consent. It should give you the basic idea of what the research is about and what your participation will involve. If you would like more detail about something mentioned here, or information not included here, please feel free to ask. Please take the time to read this carefully and to understand any accompanying information.

Background and Purpose of the Project:

It has been a very unique year since COVID-19 hit us unexpectedly in March 2020. As health professionals, we all have faced various challenges at work and in our personal lives. To abide by the social distancing public health recommendations to reduce infection risk, the delivery of primary care has significantly shifted from traditional in-person care to virtual care. This has certainly impacted your residency training in many ways. While helping sick individuals at hospitals and clinics, you may have been worried of getting infected yourself or infecting your family members. We would like to take this opportunity to listen to your learning experience. This will help us to continuously promote a better learning environment for you and future residents.

The aim of the present component of the study, in which you have been invited to participate, is to describe your learning experience during this COVID-19 pandemic. We are interested in understanding the experience related to adopting health technology to enable virtual care; primary care delivery; and resources/support for your learning and well-being needs.

Study Procedures:

You will be asked a series of questions in a focus group, which will be conducted online using the Zoom platform. The focus group should last approximately 60 minutes. The group facilitator and/or a co-facilitator will make written notes during the course of discussion. The group discussion will also be recorded.

Confidentiality:

Anything said in the discussion will be held in confidence with the facilitator(s) and principal investigators. In addition, no one other than the facilitator(s) and principal investigators will be informed of your choice to participate. All participants are also asked not to share any information about group members or the group conversation with others outside of this setting.

The written notes and video recording will be stored on the secured network drives of the Centre for Clinical Epidemiology and Evaluation in Vancouver. All network folders are password protected. The data will be kept for 5 years according to UBC policy and at the end of that period will be destroyed. No report or published paper resulting from this research will contain names, or any descriptive or other details, which might serve to identity an individual focus group participant.

Risks and Benefits:

Confidentiality precautions have been outlined above. This quality improvement study does not entail any physical adverse effects or risks to you.

You may benefit from the opportunity to share your experiences about Virtual Learning with others who are participating in the same Resident cohorts. The results may lead to program improvement and in the future contribute to a better learning for medical students undertaking Residency in British Columbia sites.

Conflict of Interest Statement:

None of the investigators has any conflict of interest to report in relation to this project.

At the time of the focus group, the moderator will answer any questions you may have before proceeding. Before or afterward, should you have any questions concerning matters related to this research or if you would like to obtain a copy of the progress reports/final report of this quality improvement study, please contact the research coordinator Demetra Barbacuta at demetra.barbacuta@ubc.ca; or the focus group facilitator Neale Smith at neale.smith@ubc.ca. If you have any concerns or complaints about your rights as a research participant and/or your experiences while participating in this study, contact the Research Participant Complaint Line in the UBC Office of Research Ethics at 604-822-8598 or if long distance e-mail RSIL@ors.ubc.ca or call toll free 1-877-822-859.

Consent:

Your participation in this study is entirely voluntary and you may refuse to participate or withdraw from the focus group at any time. If you feel uncomfortable with the nature of the questions or feel any discomfort, please inform the facilitator. If at any time you do not want to continue in the focus group, please inform the facilitator and then proceed to withdraw from group discussion by exiting the Zoom meeting.

You are encouraged to print a copy of this information for your records.

By clicking "Yes, I consent to participate in this study", you are indicating that you have understood to your satisfaction the information regarding participation in the research project and agree to participate as a subject. Also, you acknowledge that you have received a copy of this consent form. In no way does this waive your legal rights nor release the investigators, sponsors or involved institutions from their legal and professional responsibilities.

Please answer all three questions.

☐ I confirm that I am a current UBC Family Practice resident

☐ Yes, I consent to participate in this study

☐ [ ] Please type your name
